# Dietary intakes and nutritional issues in inborn errors of immunity: a systematic review

**DOI:** 10.3389/fimmu.2024.1408985

**Published:** 2024-09-27

**Authors:** Macey Freer, Rani Bhatia, Kahn Preece, Kirrilly M. Pursey

**Affiliations:** ^1^ School of Health Sciences, College of Health, Medicine and Wellbeing, University of Newcastle, Callaghan, NSW, Australia; ^2^ John Hunter Children’s Hospital, Hunter New England Health, New Lambton Heights, NSW, Australia; ^3^ School of Medicine and Public Health, College of Health, Medicine and Wellbeing, University of Newcastle, Callaghan, NSW, Australia; ^4^ Food and Nutrition Research Program, Hunter Medical Research Institute, New Lambton Heights, NSW, Australia; ^5^ Hunter New England Health, New Lambton Heights, NSW, Australia

**Keywords:** primary immunodeficiency, inborn errors of immunity, dietary intake, nutritional issues, nutritional status

## Abstract

**Introduction:**

Inborn errors of immunity (IEI) are characterized by an inherited dysregulation or absence of immune system components that can manifest clinically in complications that predispose an individual to feeding difficulties or impaired swallowing, digestion, and absorption. Treatment side-effects or altered requirements may further impair nutritional status. While adequate nutrition is necessary for optimal growth and immune function, little is known about nutritional intakes in IEI, and best practice nutrition guidelines are limited. This review aimed to synthesize current evidence on the dietary intakes, anthropometry and nutritional biochemistry in individuals with an IEI.

**Methods:**

A systematic review of literature published from database inception to March 2023 was conducted in accordance with the PRISMA guidelines. Articles eligible for inclusion reported anthropometric, biochemical, or dietary intake-related measures in pediatric or adult patients with a diagnosed IEI. Identified articles were screened for eligibility; data was synthesized descriptively.

**Results:**

A total of 4488 studies were retrieved of which 34 were included. Across studies, 2894 IEI individuals were included (age range 4 weeks to 83y), predominantly focusing on ataxia telangiectasia (AT) and common variable immunodeficiency (CVID). A significant association between inadequate energy intakes and IEI was identified (n=6 studies); however, there was significant variability in adequacy of macro- and micronutrients across studies. Patients with IEI were at risk of malnutrition (range 30% to 70%); although anthropometric assessment measures were not consistent across studies. Biochemical assessments found patients were also at risk of micronutrient deficiencies including vitamin D.

**Discussion:**

This review identified few studies assessing dietary intakes, anthropometry and nutritional biochemistry in patients with IEI, with considerable heterogeneity across studies. Future longitudinal studies using consistent validated dietary assessment tools and anthropometric measures in diverse IEI patient populations are needed. This review reinforces the need for dietetic input in people with an IEI and the development evidence-based clinical practice guidelines for people with an IEI.

**Systematic review registration:**

https://www.crd.york.ac.uk/PROSPERO, identifier CRD42023412365.

## 1 Introduction

Inborn errors of immunity (IEI), also known as primary immunodeficiency disorders (PID), are a heterogenous group of over 400 disorders characterized by an inherited dysregulation or absence of immune system components (1, 2). The prevalence of IEI globally is estimated to be up to 1% (3). These patients are characteristically vulnerable to common and opportunistic infections of increased severity and duration, which may have life-threatening consequences (3). Clinical presentations are diverse with some patients also presenting with autoinflammatory diseases, autoimmunity, bone marrow failure, allergy, and malignancy (2, 4). Symptoms often present in childhood and represent a significant health burden, impacting an individual’s quality of life and life expectancy (1, 4), and increasing risk of morbidity and premature mortality (5).

Treatments for IEI are dependent on clinical severity and the immune pathway impacted (6, 7). Milder presentations may not require treatment or may be managed with prophylactic antibiotic therapy, immunoglobulin replacement therapy, immunomodulation, or immunosuppressive therapy (6, 8). Severe and potentially life-threatening presentations, such as severe combined immunodeficiency (SCID), require hematopoietic stem cell transplantation (HSCT) (i.e. bone marrow transplant) or gene therapy to resolve genome abnormalities (6, 8). These treatments are associated with side effects that are potentially detrimental to oral intake, such as dysphagia, vomiting, diarrhea, and mucositis (9). Malnutrition has been linked to poorer outcomes post HSCT (10); enteral or parenteral nutritional support is frequently initiated to improve nutritional status and enhance treatment outcomes (10).

The role of adequate nutrition in optimizing immune function and growth has been well documented (11, 12). Impaired or altered immune function has been associated with malnutrition and nutrient deficiency, as well as overnutrition or weight gain linked to medications such as systemic corticosteroids (11, 13). A bi-directional relationship exists between malnutrition and infection (12). Malnutrition increases infection susceptibility and severity, potentially due to nutrition related immune function impairment, increasing infection related mortality. Conversely, the increased nutritional requirements and increased metabolic rate associated with infection can be detrimental to nutritional status when paired with the decreased appetite common in unwell individuals, contributing to malnutrition. Sustained inadequate dietary intake is also associated with faltering growth in children (14). These physical representations of malnutrition and impaired nutritional intake can often be the presenting features for patients with an IEI, signifying the need for further investigation (15).

Immune dysregulation associated with IEI may predispose an individual to clinical complications that can lead to feeding difficulties or impaired swallowing, digestion, and absorption (16, 17). The gut is the largest lymphoid organ and susceptible to immune dysregulation and gastrointestinal complications occur in approximately one third of patients with IEI (18, 19). These manifestations include inflammatory, structural, or oncologic disease, and are associated with chronic diarrhea, nausea, vomiting, malabsorption, weight loss and faltering growth which may or may not be infection driven (17, 18). Further impacts on nutritional status include frequent oral infections, swallowing difficulties and dysphagia, increased metabolic demands, immunosuppression, and other treatment-related side effects (6, 9, 16, 19, 20). Malnutrition is therefore prevalent within the IEI population and may require additional nutrition support prior to and during treatment (15). However, studies exploring the nutritional requirements and consequences of these treatments in IEI patients are limited.

Internationally, there are no published guidelines outlining the nutritional management of IEI. Consensus statements have been developed by prominent international bodies attempting to address major areas of uncertainty (21). However, the scope of these statements is narrow; the role of dietitian involvement in clinical care and potential nutritional manifestations is general and not explored in detail. Recent publications from national organizations in Australia have aimed to synchronize best practice management of patients with IEI. However, while the need for multidisciplinary care including a dietitian is outlined, there is limited acknowledgement of nutritional management in optimizing patient outcomes (1, 22). In addition, IEI-specific nutritional issues are not outlined, and no guidance is provided regarding the best practice nutritional management of these disorders internationally.

To date, there are no systematic reviews identifying the nutritional issues within the IEI patient population. While the importance of adequate nutrition in growth and immune function has been well documented, an understanding of nutrition within an IEI population group is vital to inform clinical practice guidelines and service delivery models (11, 12, 14). The aim of this systematic review is therefore to synthesize current evidence on the nutritional issues and dietary intakes for individuals affected by a clinically diagnosed IEI using dietary intake, anthropometric, and nutritional biochemistry measures. A secondary aim is to identify any gaps in the current literature available, to inform future research and clinical guideline development.

## 2 Methods

### 2.1 Study design

This systematic review was conducted in accordance with the Preferred Reporting Items for Systematic Reviews and Meta-Analyses (PRISMA) guidelines (23) (**Supplementary Table 1**).

### 2.2 Literature search strategy

An electronic literature search was conducted on six databases: MEDLINE, Embase, Web of Science, Scopus, CINAHL and Cochrane Library. To identify relevant human studies published in the English language from database conceptualization to current (March 2023), two sets of search terms were utilized; [1] terms related to IEI and [2] terms related to nutrition. See **Supplementary Figure 1** for example search strategy. The review protocol was registered with PROSPERO; (registration number CRD42023412365).

### 2.3 Inclusion and exclusion criteria

To be eligible for inclusion, studies had to meet the criteria outlined in **Table 1**. In brief, studies were eligible if they reported anthropometric, biochemical, or intake-related measures in pediatric or adult patients with a clinically diagnosed IEI. Studies with a comparator and with no comparator were included to understand the scope of existing literature in IEI.

**Table 1:** PECO criteria for studies assessing dietary outcomes or nutrition impact symptoms in IEI patients.

PECO Criteria	Inclusion Criteria	Exclusion Criteria
Participants	Studies were included if they included human participants of any age (i.e. pediatric or adult participants).	Pre-clinical studies
Exposure	Participants that had a clinical diagnosis of IEI/PID by an immunologist	Participants with a clinical diagnosis of secondary immunodeficiency (e.g. immunodeficiency related to cancer treatment) or self-reported IEI/PID.
Comparator	Studies were included if a non-IEI/PID group was used as a comparator. Studies without a comparator were also eligible for inclusion.	No studies were excluded due to the use or lack of comparator.
Outcome	Studies were included if they reported on one or more of the following outcomes: anthropometric outcomes (e.g. height, weight, BMI, malnutrition, growth), biochemical outcomes (e.g. lipid or micronutrient concentrations), or intake-related outcomes (e.g. energy intake, macronutrient or micronutrient intake, energy requirements, nutrition support methods).	Studies were excluded if they did not report on a minimum of one of the following outcomes; anthropometric, biochemical or intake-related outcomes.
Study Types	Randomized controlled trials, non- randomized experimental trials, case-control studies, cohort studies, cross sectional studies	Reviews, theses, commentaries, letters to the editor, case reports and studies reporting inadequate information on the methodological details of the study were not eligible for inclusion.

### 2.4 Study selection

Following the removal of duplicate citations, all studies identified in the search strategy were imported into Covidence. Titles and abstracts were screened by two independent reviewers (KMP and MF) for eligibility. Following this, full text articles were retrieved and screened for eligibility by two independent reviewers (KMP and MF). Any discrepancies in the title and abstract screening or the full text articles selected for inclusion were resolved by a third independent reviewer (RB).

### 2.5 Data extraction

A data extraction table was developed including study details (author, year, country, study design), participant characteristics (age, sex, number of participants, IEI type), anthropometric outcomes, biochemical outcomes, intake-related outcomes, and study conclusions and limitations. Data extraction was completed by one independent reviewer (MF) and checked by a second independent reviewer (KMP).

### 2.6 Quality assessment

A standardized tool, the Academy of Nutrition and Dietetics Quality Criteria Checklist for Primary Research, was used to assess the quality of retrieved studies (24). Two independent reviewers (KMP and MF) utilized this 10-item checklist to assess the risk of bias for all articles. The criteria addressed included the research question clarity; subject selection; study group comparability; withdrawal management; blinding; intervention description; outcome measure validity; suitability of statistical methods and appropriateness of data synthesis; study conclusions; and the potential for funding bias studies were assessed against the criteria. Quality items were classified as present ‘yes’ (low risk of bias), absent ‘no’ (high risk of bias), or ‘unclear’. Overall study quality was then rated as ‘Positive’ (low risk of bias), ‘Neutral’, or ‘Negative’ (high risk of bias) dependent on the presence of bias in the Quality Criteria Checklist. Any discrepancies identified during the quality assessment process were resolved by a third independent reviewer (KP or RB). All studies were included in the review irrespective of quality rating to provide an overall understanding of available literature on the topic and make recommendations about improving the quality of evidence.

### 2.7 Data synthesis

Data were synthesized descriptively. Findings were grouped according to the most common IEI types reported by retrieved studies; (i) ataxia telangiectasia (AT), (ii) common variable immunodeficiency (CVID), and (iii) other IEI diagnoses. Due to the presence of significant heterogeneity related to the nature of the studies, meta-analysis of outcomes was not possible. Therefore, data were synthesized using vote counting (25), whereby results of retrieved studies are tallied and then categorized as the following: “no association” if 0-33% studies report no association, “inconsistent” if 34-59% report a significant association, or “positive” or “negative” association if 60% or more studies report an association based on the direction of that association. If fewer than four studies report on an outcome, the association was classified as “uncertain”.

## 3 Results

### 3.1 Description of included studies

**Figure 1:**
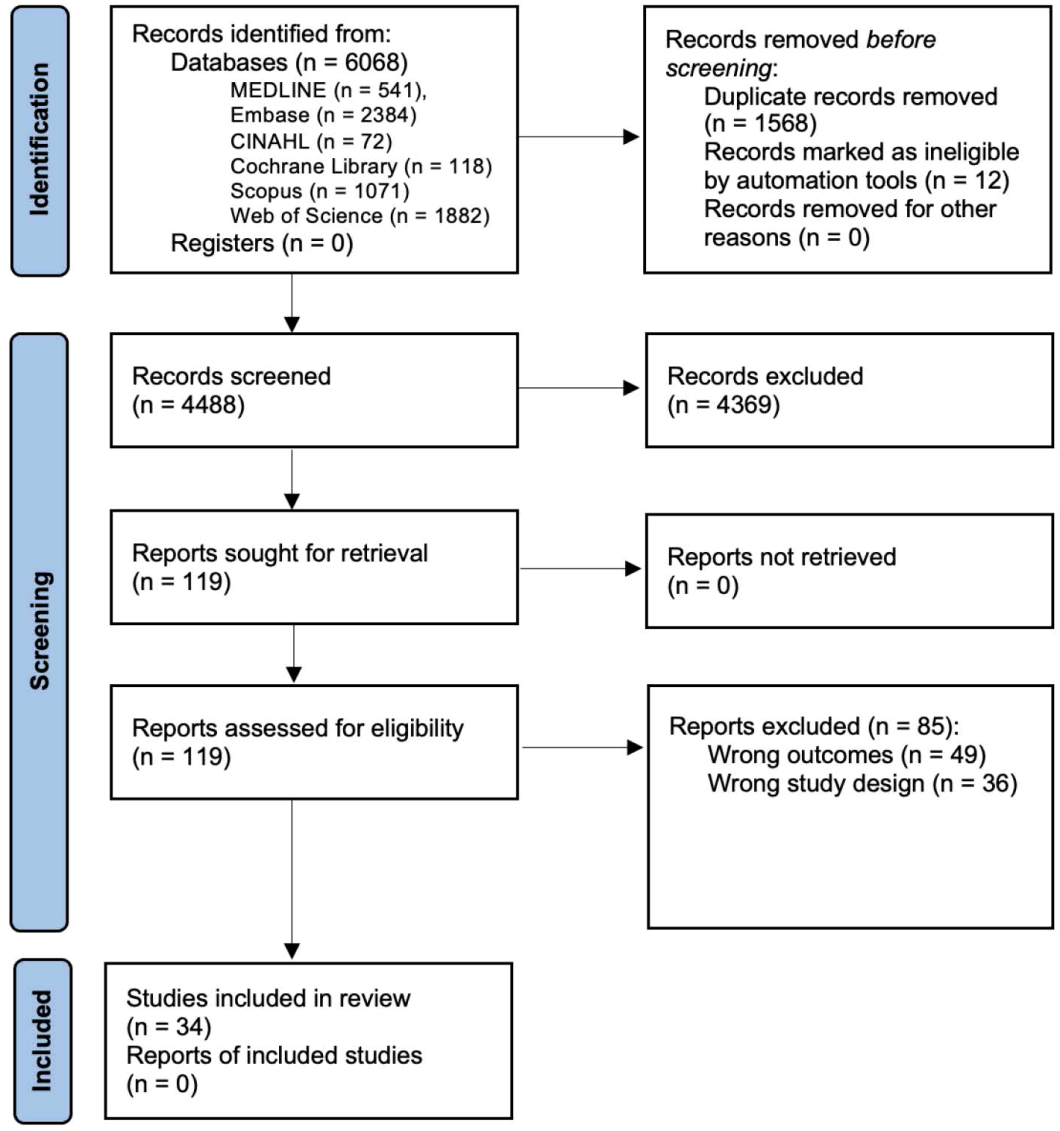
PRISMA Flow Diagram of Journal Article Identification and Inclusion or Exclusion for this Systematic Review.

A total of 4488 articles were identified of which 34 studies were included (**Figure 1**). Studies were predominantly cross sectional (n = 33) in design (10, 15, 20, 26–55); with one used a longitudinal (n = 1) design (56) (**Supplementary Table 2**).

### 3.2 Participant characteristics

A total of 3456 participants, comprising of 2894 participants with a diagnosed IEI (n = 1796 males, 1196 females, and 13 unknown) and 562 controls (range 5 to 1167 participants per study), were included across the 34 studies (**Supplementary Table 2**). Both sexes were represented in all studies, however, IEI participants were predominantly male (>50%) in 22 studies (range 50.8% - 97.3%) (4, 10, 15, 20, 26–30, 32, 41, 44, 48–56). The age of IEI participants ranged from four weeks to 83 years. Twenty-four studies reported outcomes across a range of age groups including children, adolescents, and adults; while only 10 studies reported on adult or pediatric samples separately. More specifically, seven studies were conducted in exclusively pediatric patients (4 weeks to 18 years) (10, 20, 31, 38, 50, 52, 53) and three studies exclusively adult patients (18 to 83 years) (40, 42, 45). As most studies spanned both pediatric and adult samples, and outcomes were not reported by age group, results could not be reported according to specific ages. The majority of studies were completed in AT (n = 14) (26–38) and CVID (n = 9) (39–47) patient populations. The remaining 11 studies included participants with other IEI diagnoses (SCID: n = 1 (20); primary B cell deficiency: n = 1 (50); and predominantly antibody deficiency (PAD): n = 1 (52)) or samples with different IEI diagnoses (n = 8) (10, 15, 48, 49, 51, 53–55). All studies with exclusively adult patients were in CVID populations (40, 42, 45); while studies performed with exclusively pediatric samples were completed across multiple IEI diagnoses (PAD, mixed IEI, SCID, AT, primary B cell deficiency) (10, 20, 31, 38, 50, 52, 53).

### 3.3 Quality of included studies

**Figure 2:**
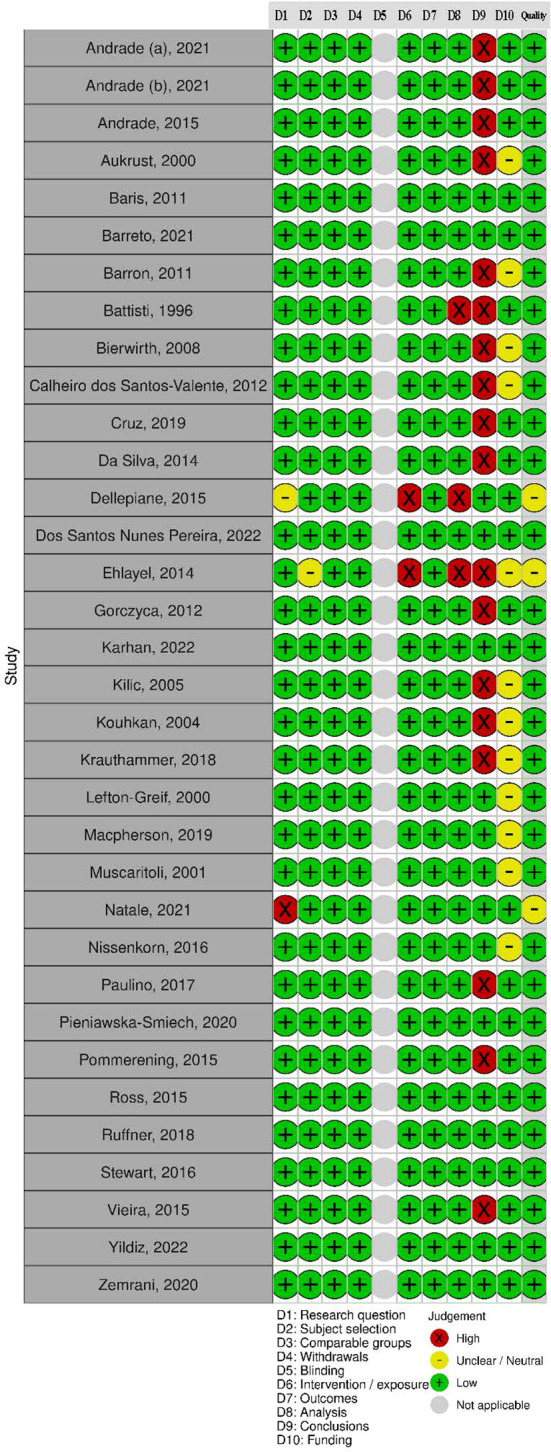
Risk of bias of included studies.

In total, 31 (91%) studies had a positive rating and three had a neutral rating (see **Figure 2**). A significant proportion of studies did not report study limitations (n =18) or disclose funding sources (n = 12). Studies rated neutral did not explicitly state the study aim or the IEI diagnostic criteria used (31, 33, 49). Additionally, there was insufficient methodological detail on statistical analysis in three studies.

### 3.4 Intake-related outcomes

Intake related outcomes were reported in 15 studies (AT: n = 8; CVID: n = 3). Nutrition and intake related outcomes, such as nutritional intake, energy requirements or supplementation, were not reported in any of the adult studies. Three pediatric only studies reported the use of enteral and parental nutrition support (10, 20, 38); one of which also found 69.2% of patients were hypermetabolic (20). Nine studies used validated assessment methods for the general population; of which three studies used food records or diaries to prospectively assess dietary intake (41, 51, 56) and six studies gathered retrospective data using 24 hour recalls (n = 5) (26, 27, 30, 35, 39) or a food frequency questionnaire (n = 1) (37). The combination of validated measures enabling the association between dietary intake and health status to be determined was only used by four studies (57) (**Supplementary Table 3**). No studies used dietary assessment tools specifically validated for people with an IEI.

#### 3.4.1 Nutrient intakes

##### 3.4.1.1 Energy

A negative association between IEI and energy intake was reported across studies using the vote counting method (25). Four studies reported low energy intakes in AT patients compared to healthy controls (27, 30) and compared to estimated energy requirements (57.8% to 64.8% EER) (37, 56), while two different studies reported no difference in energy intake compared to control (26, 35). Low energy intake was also reported in one study of CVID patients (41) and a cohort study of mixed IEI patients (51). None of these studies were reported in exclusively adult or pediatric samples.

##### 3.4.1.2 Nutrient and food intake

In people with AT, protein (27, 30, 35), carbohydrate (27, 35) and zinc (27, 30) intakes were found to be lower, however, this was not consistent across studies. One study reported lower monounsaturated fat, polyunsaturated fat, trans fat, and selenium intakes (27) in AT, while no significant difference was reported for vitamin E (26), retinol (27, 30), ascorbic acid (27), total fat (27, 35), saturated fat (27, 35), copper (27) or cholesterol (35). Only one included paper reported food groups; discretionary foods were found to contribute the largest percent energy intake in AT (37), and no studies assessed diet quality. In studies of CVID patients, lower polyunsaturated fat intakes and higher zinc and retinol intakes (26), and inadequate dietary calcium were reported (41). No significant differences were found in the one study reporting median selenium intake (39). No studies assessed dietary intake, apart from energy intake, in IEI diagnoses apart from AT and CVID. Dietary intake was also not reported in exclusively adult or pediatric samples.

#### 3.4.2 Energy requirements and nutritional support

Indirect calorimetry was used to measure resting energy expenditure (REE) in two studies (20, 37); one study reported measured REE was between 97% to 141% of the predicted basal metabolic rate in AT participants (37), while the other study reported measured REE was between 66% to 196% of the predicted REE in children with SCID (20). No studies assessed REE in adults specifically. Six studies reported using nutrition support to meet energy requirements; three of which were conducted in exclusively pediatric samples (10, 20, 38). Two studies with AT participants reported increased weight and BMI z-scores post percutaneous endoscopic gastrostomy insertion (38, 56). An additional three studies reported using a combination of oral supplementation, enteral and parenteral nutrition to meet estimated requirements in IEI participants (15, 20, 37); however, specific regimes were not reported. Few studies reported treatment side-effects in combination with dietary outcomes.

### 3.5 Anthropometric outcomes

Anthropometric outcomes were reported in 29 studies; the most common measures were BMI (n = 22), or BMI-z scores (weight, height, and BMI; n = 8, 16, and 17, respectively) (**Supplementary Table 3**). All seven studies with exclusively pediatric patients reported anthropometric measures (10, 20, 31, 38, 50, 52, 53); while no studies completed in exclusively adult patients reported anthropometric measures (40, 42, 45). In the 13 studies with AT participants reporting anthropometric outcomes spanning both children and adults, 30.8% to 69.8% of participants were classified as malnourished (n = 6) (26, 29, 30, 35, 37, 56), 31% to 44% classified as underweight (n = 4) (27, 31, 36, 38), 23.1% to 72.3% classified as short statured or stunted (n = 5) (26, 29, 31, 37, 38), 46.2% classified as wasted (37), and 4.0% to 23.1% were classified as overweight (n =4) (27, 29, 30, 35, 37). When compared to a control group, five studies found AT patients had a significantly lower body mass index (BMI) (26, 30, 35, 36, 48).

In five studies of anthropometric outcomes in adult CVID participants, between 26% to 70% were malnourished (n = 2) (46, 47), 6.3% to 36.4% were classified as underweight (n = 2) (39, 41), 46.9% were classified as overweight (n=1) (39) and 36.4% were classified as short statured (n=1) (41). No significant difference in the BMI of CVID patients was found by two studies (39, 43), however, one study demonstrated an associated between obesity and later CVID symptom development in adults (54).

In the 11 studies reporting patients with other IEI diagnoses, between 10.5% to 63.2% of children were malnourished (n = 2) (51, 52). No significant difference in malnutrition prevalence was reported between IEI diagnoses by one study (51). Across studies in varied IEI groups, between 0% to 79% of patients were classified as underweight (n = 8) (10, 20, 48, 49, 52–55), 5.3% to 60% were classified as overweight or obese (n = 6) (48, 49, 52–55), and 4.1% to 75% were classified as short statured or stunted (n = 3) (10, 20, 49).

### 3.6 Biochemistry outcomes

Biochemistry outcomes were reported in 21 studies (AT: n = 9; CVID: n = 7). All studies in exclusively adults reported biochemical outcomes (40, 42, 45); while only two studies in exclusively pediatric samples reported biochemical outcomes (31, 52). Of these studies, only one adult study reported lipids (45) and the other studies reporting micronutrient concentrations all assessed different micronutrients (31, 40, 42, 52). Thirteen reported vitamin concentrations, ten reported mineral concentrations, and ten reported lipid profiles (**Supplementary Table 3**). Dyslipidaemia was reported in between 50% to 63.6% of AT patients (27, 35) and 75% of CVID patients (39). Across all IEI groups, six studies reported altered concentrations of total cholesterol or its constituents (15, 26, 27, 39, 45, 55); while eight studies found no statistically significant difference or reported concentrations within normal parameters (27, 28, 34, 35, 39, 45, 49, 55).

#### 3.6.1 Vitamins

A negative association between IEI and vitamin D was identified (25). Five studies reported vitamin deficiencies in children and young adults with AT; 40.7% to 64% were vitamin D deficient across all five studies (31, 34, 36, 48, 56), and 23.5% of patients were vitamin K deficient (n=1) (56). However, no statistically significant difference was found across three studies in retinol (30), β-carotene (30) or serum vitamin E levels (26, 28).

Five studies reported vitamin deficiencies in child and adult CVID patients; between 20% to 36.8% of participants were found to be vitamin A deficient compared to control (n=2) (40, 44), and two studies reported 13.3% to 59.1% of participants were vitamin D deficient (n=2) (41, 48), 31.5% of participants were vitamin B6 deficient (n=1) (42), and lower retinol concentrations than control (n=1) (43). Serum folate and vitamin B12 within normal limits (n=1) (41), while another found no statistically significant difference in β-carotene concentrations in CVID compared to control (43).

#### 3.6.2 Minerals

One study reported iron deficiency in 48.7% of AT participants (56) while another found 22% had elevated serum ferritin (31). In addition, this second study reported all patients had normal serum calcium concentrations. AT patients were found to have significantly higher phosphorus by one study (48). However, no statistically significant difference in serum and erythrocyte zinc (30), serum calcium (31) or selenium concentrations (27) was reported.

In CVID participants, one study reported lower serum and erythrocyte zinc (43), while another found a significant association between CVID and selenium deficiency (39). A third study in CVID patients reported serum calcium and phosphate within normal parameters (41). High serum copper and low selenium was found in 70.3% and 37.5% of pediatric patients with predominantly antibody deficiency (PAD), while iron and zinc were predominantly within normal parameters (52).

## 4 Discussion

This is the first systematic review to assess nutritional issues and dietary intakes in IEI patients. A total of thirty-four studies were included, of which approximately 70% were conducted in AT or CVID samples exclusively. Across studies, there was a significant association between inadequate energy intake and IEI, and malnutrition was common (30-70%). However, there was significant variability in adequacy of nutrient intakes, likely due to the small number of studies investigating this, and heterogeneity of dietary intake and anthropometry assessment measures, limiting comparisons between studies. Few studies used dietary assessment tools validated for the general population to assess intake, with no tools specifically validated for IEI populations. Nutritional biochemistry reported micronutrient deficiencies in children and adults with an IEI, with a significant association between IEI and low vitamin D. This review highlights the limited research assessing anthropometric, biochemical, or dietary intake related measures of nutritional status in IEI patients, considerable variability across studies, and the underrepresentation of diverse IEI diagnoses in current literature.

This review identified a negative association between IEI and energy intake using the vote-counting method, whereby inadequate energy intakes was common in people with an IEI. Although inadequate intakes of monounsaturated fat, polyunsaturated fat, trans fat, selenium, and calcium were reported in some studies, there were insufficient numbers of studies reporting these outcomes to determine an association. In addition, no studies reported dietary intake in exclusively adult or pediatric samples, precluding conclusions about intakes in specific age groups. **Table 2** presents the recommendations for clinical practice in the identification of nutritional risk in IEI; however, these should be interpreted within the context of the limitations of existing literature. Thorough dietary assessment by clinical dietitians to determine potential deficiencies, with particular focus on these nutrients, is recommended during initial assessment and as clinically indicated. Only nine studies used dietary assessment tools validated for the general population (57), and no tools were specifically validated for IEI, highlighting the need for validated tools for use in future research. Notably, few studies in diverse IEI diagnoses assessed a range of dietary intake variables, warranting further research of dietary intakes in IEI diagnoses other than AT and CVID. As IEI includes more than 400 conditions, and with the significant heterogeneity across studies in this review, it is challenging to draw conclusions about nutrition intakes and issues across a vast and diverse group. No retrieved studies reported implementing a nutrition intervention for people with an IEI. Further studies of interactions between dietary intake and specific genotypes associated with IEI, as well as in the context of an intervention, would also advance precision nutrition for this group.

**Table 2:** Recommended dietary intake, anthropometry, and nutritional biochemistry to identify potential nutritional risks or deficiencies in people with an IEI.

Dietary assessment	Anthropometry	Nutritional Biochemistry
Energy intake Macronutrients - Protein - Dietary fats - Carbohydrate Micronutrients - Calcium - Zinc - Iron Diet quality - Food groups - Diet variety	- Height or length centile - Weight centile - BMI - BMIz for pediatrics - Malnutrition screening - Body composition (where available)	- Vitamins A, D, E, K, B6 - Iron studies - Vitamin B12 - Folate - Zinc - Selenium - Calcium - Phosphate - Serum lipids

Nutritional support was reported by six studies; however, few studies detailed specific requirements and regimens for nutrition support including oral supplements or tube feeding. Literature outlining the current practice for multidisciplinary management of AT recommends an annual nutrition assessment and early gastrostomy placement (58, 59); however, the current review identified few studies that reported nutritional intake and energy requirements in people with AT. This highlights the need for future studies that explore the types, duration, and frequency of support to further inform guidelines for nutritional support in this population. These studies would also be particularly valuable in IEI populations with elevated requirements, such as hypermetabolic patients (20), people with treatment-related side effects (60), or other diagnoses like AT where nutritional support is commonly indicated (16, 61).

Malnutrition was identified in up to 70% of IEI patients, highlighting the need for routine malnutrition screening in clinical settings and importance of nutritional management of people with an IEI. Malnutrition may cause additional detriments to people with an IEI due to its association with significant immune function impairments, such as cell-mediated immunity, phagocyte function, and cytokine production (11). The variety of anthropometric measurement techniques used and inconsistent definitions or interpretations of collected data limited finding comparability. In addition, few studies assessed anthropometry specifically in adults with an IEI. Future studies using consistent serial assessment measures across a range of age groups and diagnoses would enhance the comparability of findings and enable a more comprehensive assessment of nutritional status and malnutrition prevalence to be completed. Reporting key anthropometric outcomes according to age group (i.e. pediatric or adult) would also assist in the development of age-specific nutrition recommendations.

In this review, there was a significant association between IEI and low serum Vitamin D. Although there were an insufficient number of studies reporting a significant association with other nutritional biochemistry, this review also identified that AT patients were at risk deficiencies in Vitamin D and K, and iron, while CVID patients were at risk of deficiencies in Vitamins A, B6 and D, and selenium, and lower retinol and zinc concentrations. It is noteworthy that the most common nutritional biochemistry found to be below reference ranges in this review have documented immunomodulatory or antioxidant effects and deficiency can lead to altered immune responses, manifesting as an increased susceptibility to infection and symptom severity (11, 62). Limited studies assessed serum vitamin and mineral concentrations in people with an IEI, particularly in individuals with diagnoses other than AT and CVID. As few studies included in the review assessed the influence of supplementation on micronutrient concentrations, further interventional studies targeted at the improvement of overall diet quality or specific nutrients, or supplementation, should be considered.

Fourteen studies (41%) included participants with a diagnosis of AT, a neurodegenerative disease that has well documented nutritional complications (16, 61). A combination of involuntary movements, fatigue, and chewing or swallowing difficulties, attributed to neurological changes, can make food intake difficult and prolonged, leading to a reduced dietary intake (16). Patients with AT also present with poor growth associated with inadequate dietary intakes, frequent infections and altered hormone levels (16), and need for nutrition (16, 61). Nine studies (26%) involved participants diagnosed with CVID, which has been documented to have significant gastrointestinal involvement or autoimmune disease which can manifest in nutritional consequences such as malabsorption, malnutrition, micronutrient deficiency and growth failure (63, 64). These nutrition-related implications may explain the over-representation of these groups in the included studies.

Mixed IEI groups or less prominent IEI diagnoses were not well represented in this systematic review, particularly in the dietary intake and nutritional biochemistry domains. Future studies targeted at the identification of nutritional issues and assessment of dietary intakes for a wider variety of IEI diagnoses are warranted. Additionally, the ever-expanding worldwide availability of newborn screening for SCID, and current exploration into expanding screening programs to identify a broader range of IEI conditions (65), more pediatric patients receiving earlier HSCT with a curative intent would be expected (66). Nutritional concerns pre- and post-HSCT for IEI have been minimally explored. In addition, the current review identified few studies that explored treatment-related side effects in combination with nutrition outcomes, as well as information on autoimmune or autoinflammatory consequences in IEI. Future studies in patients receiving curative treatment are required to determine if nutritional implications are resolved post-HSCT or if new unique nutritional considerations arise. Identification of these nutritional risks would also facilitate early nutritional intervention in these patients by enabling the development of specific evidence-based guidelines.

Strengths of this review included the comprehensive and reproducible search strategy. This review was limited by the small sample sizes and cross-sectional nature of included studies, which limited the ability of data to evaluate change in outcomes over time. In addition, future studies should include a comparator group to make clear comparisons between nutrition intakes in IEI and a control group. Future longitudinal and intervention studies are required to assess change in nutrition over time, particularly in neurodegenerative conditions such as AT. The over-representation of AT and CVID diagnoses and predominantly male patients (>50% participants) in the included studies may limit the generalizability of findings to the broader IEI patient population. Nutrition-related assessment measures were highly variable and some unvalidated dietary assessment methods were used; this limited the comparability of findings and the ability to conduct a meta-analysis. In addition, quality items including acknowledging study limitations and funding sources should be reported in future research to improve the quality of the evidence base.

### 4.1 Conclusions

This review found a paucity of studies comprehensively assessing dietary intakes and nutritional issues in the IEI patient population, with considerable heterogeneity across studies. While the measures used and outcomes reported varied considerably, some key nutritional implications for IEI patients were identified, including inadequate energy intakes, low serum vitamin D, and high rates of malnutrition, underscoring the importance of dietetic input in people with an IEI. Given the nutrition-related sequelae associated with IEI and its treatment, referral to a dietitian at the point of assessment is recommended to determine risk of malnutrition, as well as conduct a thorough diet history to identify potential energy and nutrient deficiencies and provide medical nutrition therapy (**Table 2**). As this review identified potential deficiencies in nutritional biomarkers, evaluation of nutritional biochemistry during initial assessment and every 12-24 months, or as clinically indicated, is recommended to identify potential nutrient deficiencies. Vitamin and mineral supplements should be considered where nutritional deficiencies are identified, alongside clinical judgment. This review highlights future research using consistent anthropometric measures, validated dietary assessment methods, and more diverse IEI populations are needed to better understand the nutritional status and dietary intakes of IEI patients. In addition, future research should report outcomes according to age group (i.e. children, adolescents or adults) to better understand nutritional issues across the IEI lifespan and tailor clinical models of care accordingly. Longitudinal studies to track changes in nutrition over time, and intervention studies targeted at the improvement of nutritional markers and dietary intakes are also imperative. This review reinforces the need for future standardized, evidence-based guidelines for the nutritional management of IEI. The findings of this review may guide the development of consensus-based nutrition practice guidelines optimize the nutritional status of patients with IEI.

## Data Availability

The original contributions presented in the study are included in the article/**Supplementary material**. Further inquiries can be directed to the corresponding author.
