# Supplementary material for: Dietary intakes and nutritional issues in inborn errors of immunity: a systematic review
Source: Front Immunol. 2024 Sep 27;15:1408985. doi: 10.3389/fimmu.2024.1408985 (PMC11466791; doi:10.3389/fimmu.2024.1408985)
Supplement: Supplementary file 1 [file DataSheet1.docx]

Supplementary Material

# Supplementary Tables

**Supplementary Table1.** Completed 27-Item PRISMA 2020 Checklist

| **Section and Topic** | **Item #** | **Checklist item** | **Location where item is reported** |
| --- | --- | --- | --- |
| **TITLE** | | |  |
| Title | 1 | Identify the report as a systematic review. |  |
| **ABSTRACT** | | |  |
| Abstract | 2 | See the PRISMA 2020 for Abstracts checklist. |  |
| **INTRODUCTION** | | |  |
| Rationale | 3 | Describe the rationale for the review in the context of existing knowledge. |  |
| Objectives | 4 | Provide an explicit statement of the objective(s) or question(s) the review addresses. |  |
| **METHODS** | | |  |
| Eligibility criteria | 5 | Specify the inclusion and exclusion criteria for the review and how studies were grouped for the syntheses. |  |
| Information sources | 6 | Specify all databases, registers, websites, organisations, reference lists and other sources searched or consulted to identify studies. Specify the date when each source was last searched or consulted. |  |
| Search strategy | 7 | Present the full search strategies for all databases, registers and websites, including any filters and limits used. |  |
| Selection process | 8 | Specify the methods used to decide whether a study met the inclusion criteria of the review, including how many reviewers screened each record and each report retrieved, whether they worked independently, and if applicable, details of automation tools used in the process. |  |
| Data collection process | 9 | Specify the methods used to collect data from reports, including how many reviewers collected data from each report, whether they worked independently, any processes for obtaining or confirming data from study investigators, and if applicable, details of automation tools used in the process. |  |
| Data items | 10a | List and define all outcomes for which data were sought. Specify whether all results that were compatible with each outcome domain in each study were sought (e.g. for all measures, time points, analyses), and if not, the methods used to decide which results to collect. |  |
|  | 10b | List and define all other variables for which data were sought (e.g. participant and intervention characteristics, funding sources). Describe any assumptions made about any missing or unclear information. |  |
| Study risk of bias assessment | 11 | Specify the methods used to assess risk of bias in the included studies, including details of the tool(s) used, how many reviewers assessed each study and whether they worked independently, and if applicable, details of automation tools used in the process. |  |
| Effect measures | 12 | Specify for each outcome the effect measure(s) (e.g. risk ratio, mean difference) used in the synthesis or presentation of results. |  |
| Synthesis methods | 13a | Describe the processes used to decide which studies were eligible for each synthesis (e.g. tabulating the study intervention characteristics and comparing against the planned groups for each synthesis (item #5)). |  |
|  | 13b | Describe any methods required to prepare the data for presentation or synthesis, such as handling of missing summary statistics, or data conversions. |  |
|  | 13c | Describe any methods used to tabulate or visually display results of individual studies and syntheses. |  |
|  | 13d | Describe any methods used to synthesize results and provide a rationale for the choice(s). If meta-analysis was performed, describe the model(s), method(s) to identify the presence and extent of statistical heterogeneity, and software package(s) used. |  |
|  | 13e | Describe any methods used to explore possible causes of heterogeneity among study results (e.g. subgroup analysis, meta-regression). |  |
|  | 13f | Describe any sensitivity analyses conducted to assess robustness of the synthesized results. |  |
| Reporting bias assessment | 14 | Describe any methods used to assess risk of bias due to missing results in a synthesis (arising from reporting biases). |  |
| Certainty assessment | 15 | Describe any methods used to assess certainty (or confidence) in the body of evidence for an outcome. |  |
| **RESULTS** | | |  |
| Study selection | 16a | Describe the results of the search and selection process, from the number of records identified in the search to the number of studies included in the review, ideally using a flow diagram. |  |
|  | 16b | Cite studies that might appear to meet the inclusion criteria, but which were excluded, and explain why they were excluded. |  |
| Study characteristics | 17 | Cite each included study and present its characteristics. |  |
| Risk of bias in studies | 18 | Present assessments of risk of bias for each included study. |  |
| Results of individual studies | 19 | For all outcomes, present, for each study: (a) summary statistics for each group (where appropriate) and (b) an effect estimate and its precision (e.g. confidence/credible interval), ideally using structured tables or plots. |  |
| Results of syntheses | 20a | For each synthesis, briefly summarise the characteristics and risk of bias among contributing studies. |  |
|  | 20b | Present results of all statistical syntheses conducted. If meta-analysis was done, present for each the summary estimate and its precision (e.g. confidence/credible interval) and measures of statistical heterogeneity. If comparing groups, describe the direction of the effect. |  |
|  | 20c | Present results of all investigations of possible causes of heterogeneity among study results. |  |
|  | 20d | Present results of all sensitivity analyses conducted to assess the robustness of the synthesized results. |  |
| Reporting biases | 21 | Present assessments of risk of bias due to missing results (arising from reporting biases) for each synthesis assessed. |  |
| Certainty of evidence | 22 | Present assessments of certainty (or confidence) in the body of evidence for each outcome assessed. |  |
| **DISCUSSION** | | |  |
| Discussion | 23a | Provide a general interpretation of the results in the context of other evidence. |  |
|  | 23b | Discuss any limitations of the evidence included in the review. |  |
|  | 23c | Discuss any limitations of the review processes used. |  |
|  | 23d | Discuss implications of the results for practice, policy, and future research. |  |
| **OTHER INFORMATION** | | |  |
| Registration and protocol | 24a | Provide registration information for the review, including register name and registration number, or state that the review was not registered. |  |
|  | 24b | Indicate where the review protocol can be accessed, or state that a protocol was not prepared. |  |
|  | 24c | Describe and explain any amendments to information provided at registration or in the protocol. |  |
| Support | 25 | Describe sources of financial or non-financial support for the review, and the role of the funders or sponsors in the review. |  |
| Competing interests | 26 | Declare any competing interests of review authors. |  |
| Availability of data, code and other materials | 27 | Report which of the following are publicly available and where they can be found: template data collection forms; data extracted from included studies; data used for all analyses; analytic code; any other materials used in the review. |  |

*From:*  Page MJ, McKenzie JE, Bossuyt PM, Boutron I, Hoffmann TC, Mulrow CD, et al. The PRISMA 2020 statement: an updated guideline for reporting systematic reviews. BMJ 2021;372:n71. doi: 10.1136/bmj.n71 [23]

For more information, visit: <http://www.prisma-statement.org/>

**Supplementary Table 2.** Description of Included Studies

| **Author, Year, Country** | **Study Design** | **IEI Type** | **Number of IEI Participants** | **IEI Participant Characteristics – Mean Age ±** **SD or Median Age (range); % Male** | **Number of Non-IEI/Control Participants** | **Control Group Characteristics – Mean Age ±** **SD or Median Age (range); % Male** | **Anthropometry Assessment** | **Biochemistry Assessment** | **Intake-Related Assessment** | **Valid Nutrition Assessment Method Used** |
| --- | --- | --- | --- | --- | --- | --- | --- | --- | --- | --- |
| Andrade [26], 2015, Brazil | CS | AT | 13 | Mean 14.6 ± 6.2 y (range 4-24 y); 92.3% M | 22 | Mean 13.8 ± 5.9 y (range NR); 90.7% M | Weight, height, BMI, BMIz, HAZ, skinfolds (biceps, triceps, subscapular and suprailiac), body composition | Vit E, TG, TC, HDL-c, LDL-c | 1x 24HR - EEI, total fat, cholesterol, protein/lb BW, Vit E | Valid tool – repetition ± FFQ required |
| Andrade (a) [27], 2021, Brazil | CS | AT | 22 | Median 12.2 y (range 3-27 y); 72.7% M  Prophylactic ABX therapy (22.7%) | 18 | Median 15.8 y (range NR); 72.2% M | Height, weight, BMI, BMIz, HAZ, skinfolds (tricipital, subscapular, bicipital, sacroiliac), MUAC, body composition | TG, TC, HDL-c, LDL-c, plasma Se | 3x 24HR – nutrients | Yes |
| Battisti [28], 1996, Italy | CS | AT | 5 | Mean 14 ± 4.5 y (range 7-19 y); 80.0% M | 165 | Mean 11 ± 3.5 y (range NR); 80% M | Nil | Vit E, cholesterol | Questionnaire developed by authors – same dietary habits noted | No |
| Barreto [29], 2021, Brazil | CS | AT | 25 | Median 13 y (range 5-31 y);  64.0% M | N/A | N/A | Weight, height, BMI, BMIz, HAZ, skinfolds (tricipital, bicipital, subscapular, suprailiac), body composition | Nil | Nil | N/A |
| Da Silva [30], 2014, Brazil | CS | AT | 14 | Mean 13.1 ± 4.96 y (range 3-20 y); 78.6% M  Regular ABX (57.1%) | 14 | Mean 13.2 ± 4.81 y (range NR); 78.6% M | Weight, height, BMI, BMIz, HAZ, skinfolds (tricipital, subscapular), body composition, FM, LBM | Retinol, beta-carotene, serum Zn, erythrocyte Zn | 2x 24HR- EEI, protein/kg BW, retinol, beta carotene, and Zn | Yes |
| Ehlayel [31], 2014, Qatar | CS | AT | 13 | Mean 7.7 ± 3.5 y (range 3-14.5 y); sex NR | N/A | N/A | Height, weight, BMI, HtSDS | Serum Ca, P, serum ferritin, Vit D | Nil | N/A |
| Krauthammer [56], 2018, Israel | Longitudinal | AT | 53 | Mean 14.6 ± 5.2 y (range NR); 52.8% M | N/A | N/A | Weight, height, BMI, BMIz | Serum Fe, ferritin, Vit B12, folic acid, Vit D, electrolytes | Dietitian assessment – FR, EER, EEI; chart data – VFSS, PEG insertion | Valid tool – repetition ± FFQ required |
| Lefton-Greif [32], 2000, USA | CS | AT | 70 | Mean 10.7 y (range 1.8-30 y); 55.7% M | N/A | N/A | Weight, height, HAZ, WAZ, WHZ | Nil | Clinical feeding and swallowing evaluation; dietitian assessment – z-scores calculated | Unclear – insufficient detail |
| Natale [33], 2021, USA | Mixed CS & longitudinal | AT | 430 | Mean/median NR (range 6mth-34.7 y); 53.0% M | N/A | N/A | Height, weight, LAZ, HAZ, BMIz, height centiles, weight centiles, BMI centiles | Nil | Nil | N/A |
| Nissenkorn [34], 2016, Israel | CS | AT | 52 | Mean 14.1 ± 4.9 y (range 2-26.2 y); 55.8% M | N/A | N/A | Height, weight, BMI, HtSDS, WtSDS, BMI-SDS | TC, HDL-c, LDL-c, TG, Vit D | Nil | N/A |
| Paulino [35], 2017, Brazil | CS | AT | 18 | Mean 13.9 y (range 5-25 y); 83.3% M | 17 | Age NR; sex NR | Weight, height, BMI, BMIz, skinfolds (tricipital, subscapular, bicipital, sacroiliac), MUAC, body composition | TG, TC, HDL-c, LDL-c | 1x 24HR – nutrients | Valid tool – repetition ± FFQ required |
| Pommerening [36], 2015, Germany | CS interventional | AT | 25 | Mean 13.04 ± 6.79 y (range NR); 48.0% M | 26 | Mean 14.96 ± 6.45 y (range NR); 50% M | Body composition, muscle strength, weight, height, BMI, WAZ, HAZ, BMIz; bioelectrical impedance analysis - PhA, FFM, FM, BCM, ECM, ECM/BCM ratio, % BCM in FFM | Vit D | Nil | N/A |
| Ross [37], 2015, Australia | CS | AT | 13 | Mean/median NR (range 3.9-22.6 y); 38.5% M | N/A | N/A | Height, weight, height centiles, BCM, BMI, BMIz, HAZ, WHZ, BCMHZ | Nil | Dietitian interviews – diet history, FFQ, SNAQ questionnaire, mealtime assistance, tiredness/fatigue questionnaire, BMR predicted; food intake data – EEI, core food groups and discretionary choices, proportion EEI from food groups; IC - REE | Yes |
| Stewart [38], 2016, UK | Mixed CS & longitudinal with a nested case-control | AT | 101 | Median 9.3 y (range NR); 49.5% M | N/A | N/A | Height, weight, BMI, BMIz, HAZ, WAZ | Nil | Dietitian involvement – PEG insertion, nutrition support recommendations | No |
| Andrade (b) [39], 2021, Brazil | CS | CVID | 32 | Median 36.8 y (range 9.6-61.4 y); 43.8% M  Continuous ABX (43.8%) | 37 | Median 34.7 y (range NR); 43.2% M | Weight, height, BMI, BMIz, HAZ, WC | Plasma Se, TG, TC, HDL-c, LDL-c | 3x 24HR – EEI, nutrients | Yes |
| Aukrust [40], 2000, Norway | Mixed CS & longitudinal | CVID | 20 | Median 42 y (range 21-67 y); 35.0% M | 16 | Median 41 y (range 22-65 y); 37.5% M | Nil | Vit A | Vit A supplementation | N/A |
| Baris [41], 2011, Turkey | CS | CVID | 22 | Mean 14.8 ± 9 y (range 4-38 y); 72.7% M | N/A | N/A | Weight, height, WtSDS, HtSDS | Serum Ca, Serum P, Vit D, serum folate, Vit B12 | 1x 3 day FR – Ca, EEI | Valid tool – repetition ± FFQ required |
| Bierwirth [42], 2008, Germany | CS | CVID | 54 | Mean 44 y (range 21-76 y); 44.4% M | N/A | N/A | Nil | Plasma Vit B6 | Questionnaire developed by authors – diet; Vit B supplementation | No |
| Calheiro dos Santos-Valente [43], 2012, Brazil | CS | CVID | 17 | Mean 28.54 ± 11.29 y (range NR); 47.1% M | 17 | Mean 28.07 ± 10.35 y (range NR); 47.1% M | BMI | Retinol, B-carotene, serum Zn, erythrocyte Zn | Nil | N/A |
| Kilic [44], 2005, Turkey | CS | CVID | 19 | Mean 11.3 y (range 3-26.5y); 57.9% M | 15 | Mean 12.5 y (range 5-25 y); 60% M | Nil | Serum Vit A | Nil | N/A |
| Macpherson [45], 2019, Norway | CS | CVID | 102 | Mean 48 ± 15 y (range 18-83 y); 46% M | 28 | Mean 42 ± 10 y (range 28-65 y); 36% M | Nil | TC, HDL-c, LDL-c | Nil | N/A |
| Muscaritoli [46], 2001, Italy | CS | CVID | 40 | Mean 45.3 ± 16.6 y (range 17-75 y); 50.0% M | N/A | N/A | Height, weight, skinfolds (triceps), MUAC, BMI, UFA, UMA | Nil | Nil | N/A |
| Yildiz [47], 2022, Turkey | CS | CVID | 50 | Mean 40.78 ± 14.94 (range NR); 44.0% M | N/A | N/A | Screening tools - MUST, MST, SNAQ, NRS-2002; height, weight, BMI, MUAC, skinfolds (triceps), UMA | Nil | Nil | N/A |
| Barron [20], 2011, Canada | CS | SCID | 26 | Mean 4.7 ± 3.1 mth (range 0-10 mth); 76.9% M | N/A | N/A | Weight/length history, FTT, WAZ, LAZ | Nil | IC - REE; nutritional data – feeding routes to meet EER | No |
| Cruz [48], 2019, Brazil | CS | AT & CVID | 24 | Mean/median NR (range 8-56 y); 59.3% M | 24 | Age NR; 59.3% M | Weight, height, BMI, % FM, skin folds (triceps, subscapular, bicipital, suprailiac), WHR, FM, LBM | Vit D, P, Ca | Nil | N/A |
| Dellepiane [49], 2015, Italy | CS | XLA & ARA | 73 | Mean 22 ± 11 y (range NR); 97.3% M | N/A | N/A | Weight, length, height, BMI, BMIz, WAZ, LAZ, HAZ | TC, HDL-c, LDL-c, TG | Nil | N/A |
| Dos Santos Nunes Pereira [10], 2022, Brazil | Observational | Mixed IEI | 96 | Age NR; 84.4% M | N/A | N/A | Weight, height, length, BMI, BMIz, WAZ, HAZ, sum score | Nil | Medical records - alternative nutritional therapy needs | No |
| Gorczyca [50], 2012, Poland | CS | Primary B cell deficiency | 20 | Mean 8.7 ± 4.5 y (range 4-18 y); 65.0% M | RRTI: 20;  Control: 20 | RRTI: Mean 9.7 ± 4.8 y (range 4-18 y); 70% M; Control: Mean12.9 ± 3.5 y (range NR); 55% M | Height, weight, BMI, BMI centiles, height centiles, weight centiles | Nil | Nil | N/A |
| Karhan [51], 2022, Turkey | CS | Mixed IEI | 104 | Mean 9.45 ± 5.2 y (range 0.58-19.5 y); 58.7% M | N/A | N/A | Weight, length, height, WAZ, HAZ, LAZ, BMI, BMIz, pIBW | Nil | 1x 3 day FR – EEI | Valid tool – repetition ± FFQ required |
| Kouhkan [52], 2004, Iran | CS | PAD | 38 | Mean/median NR (range 2-18 y); 73.7% M | N/A | N/A | Weight, height, BMI, BMI centiles, WAZ, HAZ, WHZ | Serum micronutrients including Zn, Fe, Se, Cu | Nil | N/A |
| Pieniawska-Smiech [53], 2020, Poland | CS | Mixed IEI | 56 | Mean/median NR (range 7wks-15 y); 48.2% M | RRTI: 69;  Control: 70 | RRTI: Mean/median NR (range 11mth-16 y); 52.2% M;  Control: Mean/median NR (range 5wks-17 y); 51.4% M | Height, weight, height centiles, weight centiles, BMI, BMIz, HAZ | Nil | Nil | N/A |
| Ruffner [54], 2018, USA | CS | Mixed IEI | 1167 | Paediatric: Mean 10.1 ± 4.9 y (range 2-20 y); 67% M  Adult: Mean 41.8 ± 15.7 y (20-84 y); 58.7% M | N/A | N/A | BMI, BMI centiles | Nil | Nil | N/A |
| Vieira [55], 2015, Brazil | CS | CVID & XLA | 24 | Mean 32.1 ± 13.66 y (8-56 y); 62.5% M | 12 | Mean 29.3 ± 13.74 (range NR); 50% M | Weight, height, BMI, BMIz, HAZ, skinfolds (triceps, subscapular, biceps, suprailiac), WC, % BF, body composition | TG, TC, HDL-c, LDL-c | Nil | N/A |
| Zemrani [15], 2020, Australia | Observational | Mixed IEI | 27 | Mean/median NR (range <20 y); 74.1% M | N/A | N/A | Weight, height, WLZ, BMIz | TG, micronutrients (pre-HSCT/post-HSCT) | Medical records - nutritional support | No |

**Abbreviations:** ABX, antibiotics; ApoA1, apolipoprotein A-1; ApoB, apolipoprotein B; ARA, autosomal recessive agammaglobulinemia; AT, ataxia telangiectasia; BCM, body cell mass; BCMHZ, body cell mass for height z-scores BMI, body mass index; BMI-SDS, body mass index standard deviation scores; BMIz, BMI for age z-score; BMR, basal metabolic rate; BW, body weight; Ca, calcium; CS, cross sectional; CVID, common variable immunodeficiency; ECM, extracellular matrix; EEI, estimated energy intake; EER, estimated energy requirement; Fe, iron; FFM, fat free mass; FFQ, food frequency questionnaire; FM, fat mass; FR, food record; FTT, failure to thrive; HAZ, height for age z-score; HDL-c, high density lipoprotein cholesterol; HSCT, haematopoietic stem cell transplant; HtSDS, height standard deviation scores; IC, indirect calorimetry; IEI, inborn error of immunity; kg, kilogram; LAZ, length for age z-score; lb, pound; LBM, lean body mass; LDL-c, low density lipoprotein cholesterol; M, male; MST, malnutrition screening tool; mth, month; MUAC, mid-upper arm circumference; MUST, malnutrition universal screening tool; NR, not reported; NRS-2002, nutritional risk screening 2002; PAD, primary antibody deficiency; PEG, percutaneous endoscopic gastrostomy; PhA, phase angle; P, phosphorus; pIBW, percent ideal body weight; REE, resting energy expenditure; RRTI, recurrent respiratory tract infections; SCID, severe combined immunodeficiency; Se, selenium; SNAQ, short nutritional assessment questionnaire; TC, total cholesterol; TG, triglycerides; UFA, upper arm fat area; UMA, upper arm muscle area; VFSS, videofluoroscopic swallow study; Vit, vitamin; WAZ, weight for age z-score; WC, waist circumference; WHR, waist-hip ratio; WHZ, weight for height z-score; WLZ, weight for length z-score; WtSDS, weight standard deviation scores; XLA, X-linked agammaglobulinemia; y, years; Zn, zinc; 24HR, 24 hour food recall

**Supplementary table 3.** Outcomes of Included Studies

| **Author, Year** | **Participant Groups – IEI Group; Non-IEI/Control** | **Anthropometry Outcomes** | **Biochemistry Outcomes** | **Nutrition and Intake-Related Outcomes** | **Limitations** |
| --- | --- | --- | --- | --- | --- |
| **Studies conducted in ataxia telangiectasia cohorts** | | | | | |
| Andrade [26], 2015 | AT; control | AT patients - 30.8% malnourished and 23.1% stunted; sig. lower lean BMI in AT patients | Sig. higher TC, LDL-c, TG, and sig. lower HDL-c in AT patients; NS difference in Vit E | NS difference in energy, macronutrient, and Vit E intake; NS difference in Vit E concentrations for 46.2% AT patients regularly supplementing | Small sample size; recall bias in dietary data; recommended dietary tool combination not used |
| Andrade (a) [27], 2021 | AT; control | AT patients – 40.9% underweight and 9.1% overweight; sig. lower WC and LBM in AT group; NS difference in BMI | AT patients had sig. higher TC, LDL-c; NS difference in TG, HDL-c, and Se; 63.6% AT patients had dyslipidemia; sig. higher % inadequate LDL-c in AT patients; 59.1% adequate Se in AT patients; | Sig. lower energy, carbohydrate, protein, MUFA, PUFA, trans fat and Se intake in AT group; NS difference in Zn, retinol, ascorbic acid, total fat, saturated fat or Cu intake; AT patients – 4.5% had feeding tube, 77.3% took oral multivitamins | Small sample size; mean age NR; control age range NR |
| Battisti [28], 1996 | AT; control | Nil | NS differences in serum Vit E and cholesterol | Nil | Small sample size; dietary data NR; no valid nutrition assessment method used; p values NR; control age range NR |
| Barreto [29], 2021 | AT | 32.0% malnourished, 4.0% overweight; 42.0% short stature for age, 54.1% low LBM; 16.6% low %FM, 29.1% high %FM | Nil | 25% had dysphagia; moderate or severe ataxia in all malnourished patients | Small sample size; no control group; mean age NR |
| Da Silva [30], 2014 | AT; control | AT patients – 42.9% malnourished, 7.1% overweight; sig lower BMI, LBM in AT patients; sig. higher % malnourished and short stature AT patients | NS difference in retinol, serum Zn, erythrocyte Zn and B-carotene; sig. positive correlation between plasma retinol and serum IgA levels; NS correlation between retinol, B-carotene, serum or erythrocyte Zn with T lymphocyte numbers; NS correlations with B-carotene, serum or erythrocyte Zn | Sig. lower energy and Zn intake in AT patients; sig. higher B-carotene intake in AT patients; NS difference in protein or retinol intake; 50.0% AT patients supplementing Vit regularly – NS association to micronutrient concentrations | Small sample size; recall bias in dietary data; control age range NR |
| Ehlayel [31], 2014 | AT | 38% stunted, 31% underweight | 61.5% Vit D deficient, 22% elevated serum ferritin, 100% normal serum Ca and P | Nil | Small sample size; no control group; sex NR |
| Krauthammer [56], 2018 | AT | 61.4% ≥ one measurement of severe malnutrition, only 15.9% had a normal nutritional state; BMIz sig. inversely correlated with age – decline in centiles observed in M >4 y, F >7 y, 17.0% within normal limits > 15 y | 48.7% Fe deficient, 38.8% anaemic, 40.7% Vit D deficient, 23.5% Vit K deficient | Average caloric intake 64.8% F and 57.8% M EER; sig. decrease in relative % caloric intake with age; weak positive correlation between BMIz and % caloric intake; significant BMIz score increase post-PEG insertion; one patient died 3 weeks post-PEG insertion due to complications of severe malnutrition; 100% received nutritional counselling – no long term improvement; 67.9% coughing/choking during meals – 58.3% had abnormal swallow | Small sample size; no control group; missing or incomplete measurement (17%) and food diary (55%) data; recommended dietary tool combination not used; age range NR |
| Lefton-Greif [32], 2000 | AT | Compared to clinical only group, VFSS patient group had sig. lower height, weight and WHZ; sig. lower mean WAZ and WHZ in aspirating patients; lower than normative growth parameters at all ages | Nil | 27% patients aspirated ≥ one swallow – sig. higher mean age; 71.4% aspirating patients failed to demonstrate clinical signs of aspiration | Small sample size; no control group; no valid nutrition assessment method reported |
| Natale [33], 2021 | AT | AT median BMI further below CDC median with increasing age; F height faltering sooner/more severe; 88% F and 62% M with classic AT height <10^th^ centile by adulthood; 67% F and 41% M with classic AT ≥ 13 y stunted, 3.8% of mild AT patients ≥ 13 y stunted; proportion malnourished patients increased with age, 5.5% classic AT severely malnourished; no overweight adult classic or mild AT patients, classic AT most frequently overweight during ages 3-5 y | Nil | Nil | No control group; incomplete or missing anthropometric data; mean age NR |
| Nissenkorn [34], 2016 | AT | Median HtSDS negative at all ages, more impaired than WtSDS in infancy. WtSDS and BMISDS gradually decreased; more prominent growth retardation in F during later childhood/adolescence | Mean TC, HDL-c, LDL-c and TG within normal parameters; 9.6% had ≥ one LDL-c measurement > 130mg/dl, 15.4% had ≥ TG measurement > 130mg/dl; 23.1% Vit D deficient and 9.6% Vit D insufficient | Nil | Small sample size; no control group; missing data, patients not recalled or deceased; varying number and timing of measurements |
| Paulino [35], 2017 | AT; control | AT patients - 33.3% malnourished, 5.6% overweight, 55.6% low MUAC, 11.1% low FM and 38.9% high FM; sig. lower mean BMI in AT patients | NS difference in TC, LDL-c, TG, and HDL-c; 55.5% AT patients had dyslipidemia | NS difference in energy, protein, carbohydrate, total fat, saturated fat, cholesterol intake | Small sample size; recall bias in dietary data; recommended dietary tool combination not used; control age/sex NR |
| Pommerening [36], 2015 | AT; control | AT patients – 44% underweight; sig. lower height, weight, BMI, PhA, FFM, BCM, ECM, % BCM in FFM, manual muscle strength and sig. higher ECM/BCM ratio in AT patients; 33.3% AT patients had pathologically low PhA values | Sig. lower Vit D in AT patients >12 y compared to controls; 64% Vit D deficient – 33.3% wheel-chair bound AT patients Vit D deficient | 8.0% AT patients had gastrostomy tubes; 66.7% wheelchair-bound AT patients had dysphagia | Small sample size; age range NR |
| Ross [37], 2015 | AT | 23.1% short stature, 69.2% stunted, 46.2% wasted, 7.7% severely underweight, 23.1% overweight or obese, 69.2% malnourished from BCMHZ; sig. relationship between age and increasing malnutrition | Nil | REE between 97% to 141% predicted BMR; EER met or exceeded by 38.5% of participants; 61.5% consumed less energy than requirements – 37.5% of participants consuming inadequate energy were overweight or obese; sig. correlation between REE and BCM/height; most energy from discretionary foods, grain then meat largest energy portion of core food groups, low fruit and vegetable consumption; 15.4% patients had supplementary overnight feeds supplying 44% to 56% of their energy intake; nutrition issues – 15% poor appetite, narrow food range, fatigue, need for meal assistance | Small study size; no control group; recall bias in dietary data; mean age NR |
| Stewart [38], 2016 | AT; control | 34.7% underweight and 72.3% stunted ≥ one occasion; sig. decline of WAZ by 0.1 units/y – 0.1 SDS decrease annually compared to healthy population; non-linear weight, height and BMIz | Nil | 40.0% of underweight patients for whom longitudinal data were available had PEG in situ – WAZ improved in 70.0%; 75.0% had improved WAZ post-PEG; sig. more AT patients weight increased post-PEG insertion | Small sample size; no valid nutrition assessment method; mean age NR |
| **Studies conducted in common variable immunodeficiency cohorts** | | | | | |
| Andrade (b) [39], 2021 | CVID; control | CVID patients - 6.3% underweight, 46.9% overweight and 37.5% high WC; NS difference in BMI | Sig. lower median Se in CVID patients; NS difference in TC, LDL-c, HDL-c, and TG; 75.0% had dyslipidemia and 50% had inadequate Se in CVID patients; sig. lower plasma Se in patients with chronic diarrhea | Sig. lower PUFA and sig. higher Zn and retinol intake in CVID patients; NS difference in median Se intake; 21.9% regularly supplementing with vitamins or food | Small sample size; recall bias in dietary data; control age range NR |
| Aukrust [40], 2000 | CVID; control | Nil | Sig. lower Vit A in CVID patients; 20% Vit A deficient in CVID patients; Vit A supplementation in patients with low levels resulted in a sig. increase vs baseline in plasma Vit A levels at 3mth and 6 mth measures | Supplementation in low Vit A patient increased IL-10 and IgA levels, and decreased TNF$\alpha$ and neopterin | Small sample size; mean age NR |
| Baris [41], 2011 | CVID | 36.4% short stature, 36.4% underweight | 59.1% Vit D deficient; serum Ca, P, serum folate, Vit B12 within normal limits; BMDz at lumbar spine/femoral neck sig. correlated with serum folate levels | Inadequate dietary Ca and low caloric intake in 77.3% of patients; lower femur z-scores associated with lower caloric intake | Small sample size; no control group; recommended dietary tool combination not used |
| Bierwirth [42], 2008 | CVID | Nil | 31.5% Vit B6 deficient 100% deficient patients supplementing Vit B6 had normal levels after 3mth | Sig. increase in CD4+ lymphocytes after Vit B6 substitution; NS improvement in serum immunoglobulin concentrations after Vit B6 substitution. | Small sample size; dietary data and BMI NR; no control group; no valid nutrition assessment method |
| Calheiro dos Santos-Valente [43], 2012 | CVID; control | NS difference in BMI | Sig. lower retinol, serum Zn, erythrocyte Zn in CVID group; NS difference in B-carotene | Nil | Small sample size; age range NR |
| Kilic [44], 2005 | CVID; control | Nil | Sig. lower mean serum Vit A in CVID patients, 36.8% Vit A deficient; sig. increased TNF-α, IL-4 and IL-2 in cultured mononuclear cells obtained from CVID patients post in vitro Vit A supplementation |  | Small sample size |
| Macpherson [45], 2019 | CVID; control | Nil | Sig. lower plasma HDL-c in CVID patients; sig. lower HDL-c in CVID patients with non-infectious complications vs infections only; NS difference in LDL-c and TC; functional studies - impaired HDL cholesterol acceptor function and reverse cholesterol transport from macrophages in CVID patients | Nil | Small sample size |
| Muscaritoli [46], 2001 | CVID | Sig. higher FM and FFM depletion in CVID patients compared to normal population; higher incidence of FFM depletion in M participants; higher FM depletion frequency in F or patients with diarrhoea; 30.0% BW lower than IBW, 30.0% malnourished; higher prevalence of morbidity and recurrent infections in patients with an impaired nutritional status | Patients with lower CD4+ T cells and undetectable IgA had higher nutritional impairment; no malnourished patients had detectable IgA | Nil | Small sample size; no control group |
| Yildiz [47], 2022 | CVID | Malnutrition risk from screening tools - 48% MUST, 26% MST, 20% SNAQ, 20% NRS-2002; 54% malnourished according to low MM and 30% according to low BMI; malnutrition identified in 70% of the patients classified as at risk for malnutrition according to the MUST score | Lower IgA and lower CD19+ B-cell counts sig. associated risk factors for malnutrition | Nil | No control group; age range NR; collected anthropometry data NR |
| **Studies conducted in other inborn errors of immunity cohorts** | | | | | |
| Barron [20], 2011 | SCID | 57.7% had FTT: 60% of those had ≥ two centiles between weight/height, 33.3% underweight and stunted, and 7% were stunted with documented FTT; sig. association between infection and FFT; diarrhea sig. more common in FTT patients | Nil | 69.2% hypermetabolic, 93.3% FTT patients hypermetabolic; sig. positive association between FTT and hypermetabolism; sig. more hypermetabolic infants at 3-12mth vs <3 mth; mean measured REE sig. higher than mean predicted REE – measured REE between 66% to 196% of predicted REE; mean 25 days required to meet hypermetabolic patient energy needs – 35.3% oral route, 23.5% EN, 52.9% PN, 50% EN patients had supplemental PN; EN/PN not required by patients with normal metabolic rates | Small sample size; no control group; incomplete data; no valid nutrition assessment method |
| Cruz [48], 2019 | AT & CVID; control | Compared to control, sig. higher LBM in CVID patients; sig. lower BMI, LBM, FM, WC and WHR in AT group; NS difference in all other measurements; 0% underweight and 60% overweight in CVID patients, 66.6% underweight in AT patients; sig. negative correlation between Vit D and FM in CVID patients and BMI or % total fat in AT patients; NS correlation between Vit D and other anthropometric measures | NS difference in Vit D levels between CVID and AT patients; 44.4% AT and 13.3% CVID Vit D deficient, 22.2% AT and 53.3% CVID Vit D insufficient; sig. higher P in the AT group compared to control; NS difference in Ca levels; NS correlation between Vit D and Ca or Vit D and P | Nil | Small sample size, Vit D sample collections performed during different seasons; IEI mean age NR; control age/sex NR |
| Dellepiane [49], 2015 | XLA & ARA | 5.5% underweight, 38.4% overweight or obese, 4.1% stunted; 13.6% underweight and 45.5% overweight or obese at diagnosis | TC, HDL-c, LDL-c, and TG within normal parameters | 1.4% using PN | Small sample size, no control group; age range NR |
| Dos Santos Nunes Pereira [10], 2022 | Mixed IEI | SCID patients - 60.0% low WAZ, 52.0% short stature and 44.0% with thinness; HS patients - 75% short stature and 100% normal or overweight; variation in nutritional status in other IEI – majority adequate nutrition in WAS and PSD patients; SCID patients had sig. lower WAZ and BMIz compared to WAS/HS patients and HAZ compared to WAS patients; NS association between anthropometry measurements and 6mth survival outcomes or aGVHD occurance | Nil | 46.9% EN and 17.7% PN used by patients for alternative feeding routes | Small sample size; no control group; no valid nutrition assessment method; age NR |
| Gorczyca [50], 2012 | Primary B cell deficiency; RRTI & control | Compared to controls, sig. lower BMI values and weight and height centiles in IEI group – NS differences compared to RRTI group | Nil | Nil | Small sample size |
| Karhan [51], 2022 | Mixed IEI | 42.3% ≥ one measurement of malnutrition, 21.2% severely malnourished; 48.9% CID, 32.1% PAD, 58.8% PSD malnourished; highest malnutrition rates in CID less profound than SCID (52%), CGD (66.6%), and XLA (50%); 22.1% acute malnutrition from BMIz, 27.9% malnourished from pIBW; NS difference in malnutrition type or prevalence between IEI groups | Nil | Daily caloric intake ≤ 20% requirements in malnourished patients | Small sample size; no control group; recommended dietary tool combination not used |
| Kouhkan [52], 2004 | PAD | 21.1% underweight and 10.5% overweight or obese from BMI; 63.2% HAZ malnourished; 50.0% WAZ malnourished; 15.8% WHZ malnourished and 5.3% WHZ overweight; sig. higher malnutrition frequency compared to CDC standard | 70.3% high serum Cu, 37.5% (n = 15) low Se; Fe and Zn normal for most patients | Nil | Small sample size; no control group; mean age NR |
| Pieniawska-Smiech [53], 2020 | Mixed IEI; RRTI & control | Almost 20% IEI patients underweight during hospitalization – sig. more patients underweight in IEI group, sig. more IEI children in lower weight centiles; 17.8% IEI patients stunted during hospitalization – sig. difference in height for certain centiles between patient groups and HAZ between sexes in IEI patients; 23.7% underweight and 7.1% overweight in IEI group; sig. lower nutritional status in IEI patients; NS difference in BMIz scores; sig. lower birth weight in IEI patients | Nil | Nil | Small IEI sample size; mean ages NR |
| Ruffner [54], 2018 | Mixed IEI | 25.8% obese and 5.6% underweight in adult IEI patients, 13.2% obese and 6.6% underweight in pediatric IEI patients; NS difference in obesity prevalence between databases (except 1 year in USIDNET adult cohort); sig. increased underweight prevalence across multiple years in USIDNET adult cohort; 79% underweight adults CVID patients; sig. association between later symptom development and obesity in CVID patients | Nil | Nil | Distribution of PID diagnoses in cohort varies from the USIDNET population; no longitudinal data; no control group |
| Vieira [55], 2015 | CVID & XLA; control | NS difference in BMI, WC and % BF; IEI patients - 12.5% underweight and 33.3% overweight | Sig. lower HDL-c in IEI patients; NS difference in TG, TC, and LDL-c; sig. association between low HDL-c and higher BMI, WC and TG in IEI patients | Nil | Small sample size; control age range NR |
| Zemrani [15], 2020 | Mixed IEI | 33.3% had faltering growth, 33.3% children underweight and/or stunted pre-HSCT 33.3% patient weights and 22% patient heights > 50^th^ percentile pre-HSCT; moderate WAZ/HAZ decrease at 1mth and 3mth post-HSCT, progressive catch up growth from 6mth post-HSCT, WAZ exceeded pre-transplant values at 1 year; 8.3% underweight and 16.7% stunted at 1 year post-HSCT – all had upward trending data from pre-HSCT baseline | 55.6% patients had high TG post-transplant | 33.3% had EN pre-conditioning therapy, 18.5% had gastrostomy inserted and 7.4% had prolonged PN pre-HSCT due to intestinal failure; 18.5% exclusive EN post-HSCT, 81% required supplemental or exclusive PN within 12/12 post-HSCT, 11.1% received no EN due to absent NGT; 59.3% had EN >100 days post-HSCT, 51.8% had PN >30 days, 33.3% had multiple episodes of PN. – nutritional rehabilitation increased weight, height and BMI in undernourished patients; | Small sample size; no valid nutrition assessment method; mean age NR; no control group |

**Abbreviations:** aGVHD, acute graft versus host disease; ARA, autosomal recessive agammaglobulinemia; AT, ataxia telangiectasia; BCM, body cell mass; BCMHZ, body cell mass for height z-scores; BF, body fat; BMI, body mass index; BMI-SDS, body mass index standard deviation scores; BMIz, BMI for age z-score; BMR, basal metabolic rate; Ca, calcium; CDC, centers for disease control and prevention; CID, combined immunodeficiencies; chronic granulomatous disease; Cu, copper; CVID, common variable immunodeficiency; DEXA, duel-energy X-ray absorptiometry; ECM, extracellular matrix; EER, estimated energy requirement; EN, enteral nutrition; F, female; Fe, iron; FFM, fat free mass; FM, fat mass; FTT, failure to thrive; HAZ, height for age z-score; HDL-c, high density lipoprotein cholesterol; HS, hidradenitis suppurativa; HSCT, hematopoietic stem cell transplant; HtSDS, height standard deviation scores; IEI, inborn error of immunity; IgA, immunoglobulin A; IL-4, interleukin-4; IL-10, interleukin-10; LBM, lean body mass; LDL-c, low density lipoprotein cholesterol; M, male; MM, muscle mass; MST, malnutrition screening tool; mth, month; MUAC, mid-upper arm circumference; MUFA, monounsaturated fatty acid; MUST, malnutrition universal screening tool; NR, not reported; NRS-2002, nutritional risk screening 2002; NS, not statistically significant; PAD, primary antibody deficiency; PN, parenteral nutrition; PEG, percutaneous endoscopic gastrostomy; PhA, phase angle; P, phosphorus; pIBW, percent ideal body weight; PSD, phagocytic system disorders; PUFA, polyunsaturated fatty acid; REE, resting energy expenditure; RRTI, recurrent respiratory tract infection; SCID, severe combined immunodeficiency; Se, selenium; sig., statistically significant; SNAQ, short nutritional assessment questionnaire; TC, total cholesterol; TG, triglycerides; TNF$\alpha$, tumor necrosis factor-$\alpha$; VFSS, video fluoroscopic swallow study; Vit, vitamin; WAS, Wiskott-Aldrich Syndrome; WAZ, weight for age z-score; WC, waist circumference; WHR, waist-hip ratio; WHZ, weight for height z-score; WtSDS, weight standard deviation scores; XLA, X-linked agammaglobulinemia; y, years; Zn, zinc

# Supplementary Figures


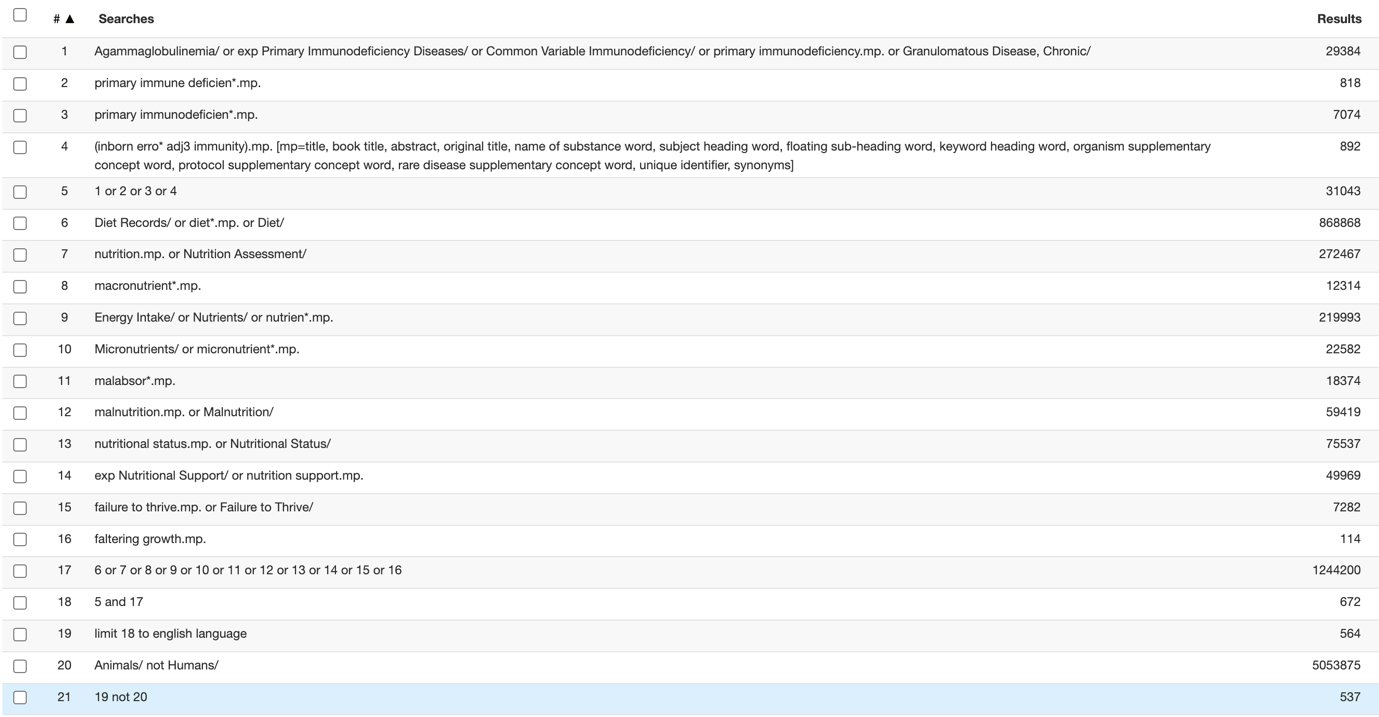


**Supplementary Figure.** Search Strategy for the MEDLINE Database
